# Upper eyelid retraction: meta-analysis & systematic review of surgical outcomes

**DOI:** 10.1016/j.jpra.2026.09.018

**Published:** 2026-09-16

**Authors:** Lorenzo Governatori, Beniamino Brunetti, Paolo Persichetti, Anastasia Fidanza, Alessandra Scampoli, Michele Pascali, Francesco Paolo Bernardini, Giovanni Francesco Marangi, Stefania Tenna, Tomaso Caporossi

**Affiliations:** a Department of Plastic, Reconstructive and Aesthetic Surgery, Fondazione Policlinico Universitario Campus Bio-Medico, Rome, Italy; b Ophthalmology Unit, "Fatebenefratelli Isola Tiberina Gemelli Isola Hospital", Rome, Italy; c Catholic University "Sacro Cuore", Rome, Italy; d Independent Plastic and Reconstructive Surgeon, Rome, Italy; e Research Unit of Plastic, Reconstructive and Aesthetic Surgery, Università Campus Bio-Medico di Roma, Rome, Italy; f Plastic Surgery Academy, Rome, Italy; g Oculoplastica Bernardini, Genova/Milan, Italy

**Keywords:** Upper eyelid retraction, Meta-analysis, Müller recession, Levator recession, Blepharotomy, Spacer graft

## Abstract

**Background:**

Upper eyelid retraction (UER) is the hallmark of thyroid eye disease and a recognized complication of aesthetic and reconstructive eyelid surgery. Multiple levator-Müller targeted procedures exist, yet technique selection remains empirical, and no prior quantitative synthesis focused on posterior-lamellar lengthening has been available.

**Methods:**

A PRISMA-guided systematic review and single-arm meta-analysis was performed. Studies reporting MRD1 (Marginal Reflex Distance 1) change, anatomical success, or reoperations rates after retractor-lengthening surgery were included. Subgroup and meta-regression analyses assessed baseline severity, surgical macro-area, approach, etiology, and spacer material. Risk of bias was evaluated using ROBINS-I and RoB 2.

**Results:**

Thirty-nine studies comprising 2064 patients (3096 eyelids) were analyzed. Pooled ΔMRD1 was 2.71 mm (95% CI 2.36-3.06) and anatomical success 85% (95% CI 80.6-89.1%). Preoperative severity explained most heterogeneity (β = 0.72 mm/mm; p < 0.0001), while surgical technique had no independent effect after adjustment. Reoperation rate was 12% (95% CI 9.1-15.5%), with increased odds after levator-only procedures (OR 3.5; p = 0.014) and levator/Müller recession-resection (OR 2.5; p = 0.034). Autologous spacers provided higher success (88%) and fewer reoperations (5%) than non-autologous grafts (76%; 25%). Overcorrection was more frequent with levator-only techniques (OR 5.6; p = 0.020), whereas undercorrection correlated inversely with baseline severity (β = -0.33; p = 0.045).

**Conclusions:**

Surgical correction of UER yields reliable outcomes when matched to disease severity. Baseline retraction, rather than technique, predicts postoperative eyelid position, while reoperation risk differentiates procedures, favoring blepharotomy and autologous spacers. These findings support a severity-matched approach for surgical planning and patient counseling.

## Introduction

Upper eyelid retraction (UER) represents a surgical challenge between function and aesthetics. The abnormal superior displacement of the upper eyelid margin exposes the ocular surface, increases the risk of exposure keratopathy and potential blindness,^1^ while producing a characteristic “staring” appearance that markedly affects quality of life^1^^,^^2^ (Supplemental Fig. 1). Although most commonly associated with thyroid eye disease (TED), where it affects up to 90% of patients,^3^ UER also arises from iatrogenic causes,^4^^,^^5^ trauma or scarring sequelae,^5^^,^^6^ facial nerve palsy or myogenic disorders,^5^, ^6^, ^7^, ^8^ and these non-TED etiologies share the same surgical correction principles.^6^^,^^9^

Effective management requires identifying whether the abnormality lies in the anterior lamella (skin, orbicularis muscle), middle lamella (orbital septum and fat) or posterior lamella (Müller/Levator muscle complex, conjunctiva).^10^^,^^11^ Posterior surgical strategies focus on weakening or lengthening the Müller muscle and levator palpebrae superioris through anterior or posterior approaches, with or without spacer grafts. Over the past three decades, numerous modifications have been proposed, yet outcomes remain heterogeneous, partly owing to variations in technique selection, surgeon experience, and inconsistent definitions of anatomical success.^8^^,^^12^^,^^13^

Despite several narrative reviews,^1^^,^^14^^,^^15^ there is no unified quantitative synthesis of the functional and anatomical outcomes of retractor-lengthening procedures. Recently, a scoping review has comprehensively mapped medical and surgical treatments for Graves-associated upper eyelid retraction^16^; however, its etiologic restriction and descriptive design precluded quantitative synthesis or identification of surgical outcome determinants. This gap limits evidence-based counselling across techniques. The aim of this meta-analysis is to provide an organized, updated synthesis of levator and Müller muscle lengthening procedures.

## Methods

This review followed PRISMA guidelines (Fig. 1, Supplemental Table 1) and included adult patients with upper eyelid retraction (UER) of any etiology. Eligible studies evaluated surgical techniques aimed at weakening or lengthening the upper eyelid retractors, including levator recession/myotomy, Müller muscle excision or recession, combined procedures, spacer graft techniques, and full- or partial-thickness blepharotomy. As this was a single-arm meta-analysis, no direct comparator was required; subgroup analyses by technique and etiology were planned a priori. Procedures were classified into six predefined macro-areas: Blepharotomy, Levator/Müller, Levator-only, Müller-only, Spacer graft, Combined/Hybrid (Supplemental Table 2). This systematic review and meta-analysis was conducted using data from previously published studies. As no original patient data were collected, institutional review board (IRB) approval was not required.

**Fig. 1:**
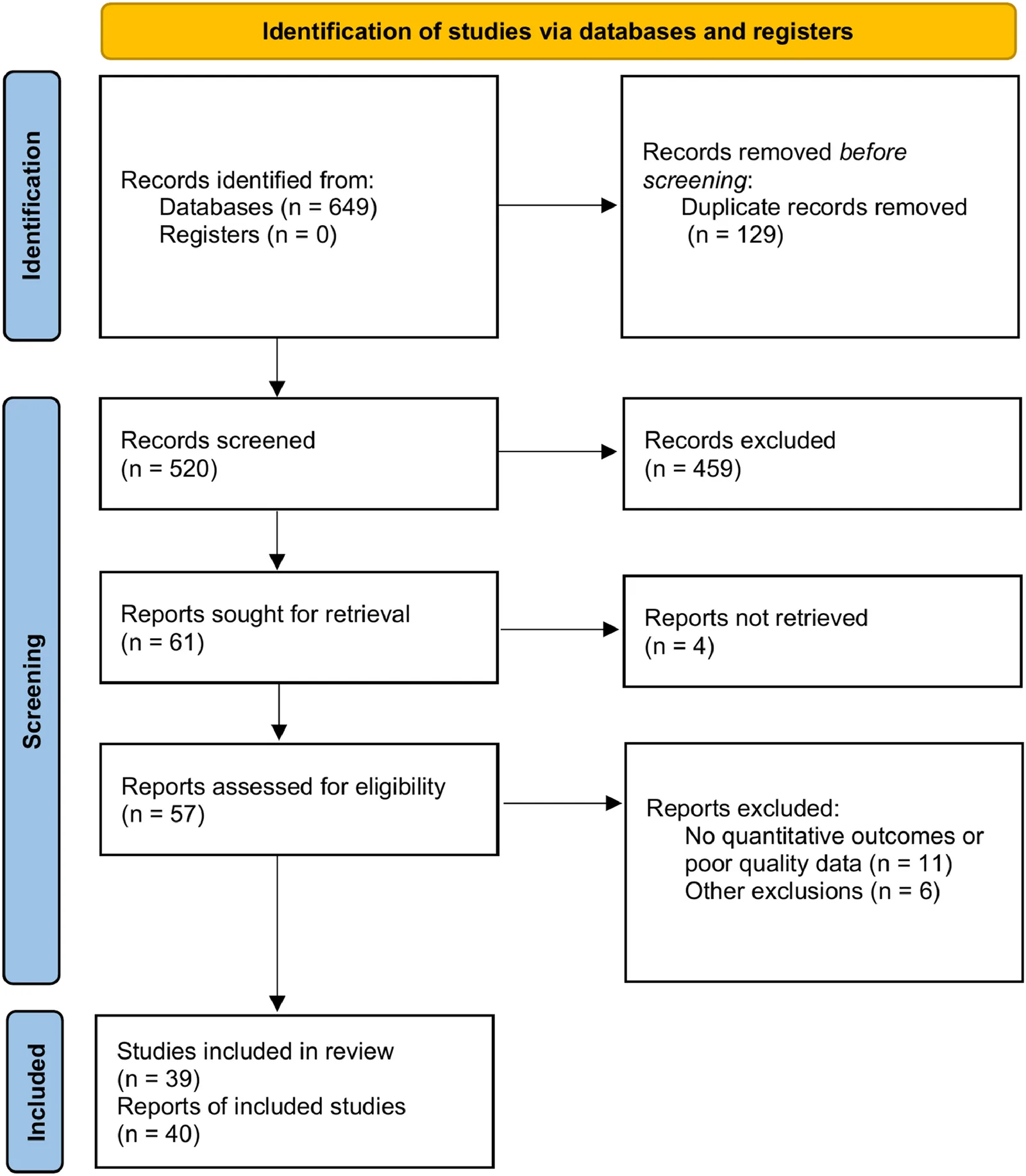
PRISMA 2020 flow diagram for new systematic reviews which included searches of databases and registers only. (Source: Page MJ, et al. BMJ 2021;372:n71. Licensed under CC BY 4.0).

Primary outcomes were mean change in marginal reflex distance 1 (ΔMRD1) and surgical success. Because several studies used alternative measures (palpebral fissure height, scleral show, retraction above the limbus), standardized conversions were applied to harmonize outcomes onto an MRD1 scale (Supplemental Table 2). Surgical success definitions varied across studies and were harmonized based on data availability according to the principles described by Mourits and Sasim,^17^ with anatomical success defined as postoperative MRD1 3-5 mm and/or inter-eyelid asymmetry ≤1 mm, with a smooth and regular eyelid contour. Secondary outcomes included under/overcorrection, reoperation, and contour abnormalities.

Studies were eligible if they reported quantitative outcomes, included ≥5 patients/eyelids, and had a mean follow-up of at least 3 months. Case reports, abstracts, non-English publications, and studies lacking analyzable data were excluded. Two reviewers independently extracted study characteristics and outcomes. When required, means and standard deviations were derived from medians and ranges using validated transformation methods by Hozo et al., Wan et al., and Luo et al. Missing SDs for ΔMRD1 were imputed using prespecified correlation coefficients (r = 0.2, 0.5, 0.8), enabling sensitivity analyses.

When a single study reported multiple technique-specific cohorts, these were analyzed separately. This approach accounts for the discrepancy between the number of included studies and the number of cohorts analyzed for individual outcomes. Risk of bias was assessed using RoB-2 for randomized trials and ROBINS-I for observational studies.

Analyses were performed in R. Subgroup analyses were performed according to surgical macro-area, surgical approach, and etiology (TED vs non-TED). Binary outcomes were pooled using logit-transformed proportions with inverse-variance random-effects models (Hartung-Knapp; ML estimator). Continuous outcomes were synthesized using random-effects models (REML; Hartung-Knapp). Heterogeneity (τ², I², Q) and 95% prediction intervals were reported. Leave-one-out analyses were conducted and publication bias was examined with funnel plots and Egger regression when ≥10 cohorts were available. Meta-regression analyses were performed to explore moderators of ΔMRD1 and success rates, using predefined covariates including baseline eyelid position, surgical macro-area, and etiology. The protocol was prospectively registered in PROSPERO (CRD420251134333).

## Results

### Study selection

A literature search of PubMed/MEDLINE, Scopus, and Cochrane identified 649 records (Supplemental Table 2). After removing 129 duplicates, 520 were screened; 459 were excluded at title/abstract level. Of 61 full-text articles assessed, 4 could not be retrieved. 57 were assessed for eligibility, of which 17 were excluded. The final dataset comprised 39 studies (40 reports) (Fig. 1).

### Baseline characteristics and study population

Thirty-nine studies (30 retrospective, 7 prospective, 2 RCTs) comprising 2064 patients (3096 eyelids) were analyzed (Table 1). The cohort showed female predominance (77%), with TED as the primary etiology (78.4%) and iatrogenic retraction second (17.7%). Mean age was 51.2±4.3 years. Preoperative MRD1 averaged 6.5±1.3 mm. Anterior approaches predominated (82.8%). Levator/Müller recession was most frequent (35.5%), followed by spacer-graft (19.1%), levator-only (17.3%), blepharotomy (15.0%), combined/hybrid (8.1%), and Müller-only (5.0%). Numbers of cohorts vary slightly across outcomes due to exclusion of techniques with insufficient data for specific analyses (see table footnotes)

**Table 1:** Baseline characteristics.

Study	Design	Patient/Eyelid	Female patient, (%)	Age, y mean ± SD	Retraction Etiology	Upper Eyelid Lengthening Surgical Technique G1/G2	Approach	Follow up, mo mean ± SD or range	MRD1 pre, mm mean ± SD
Park 2025	Retrospective	64/70	57 (89)	NA	Iatrogenic	Homologous Spacer (AlloDerm®)	Anterior	1-18	6.5 ± 1*
Gonçalves 2017	RCT	15/27 12/20	19 (70)	42.2 ± 9.0	TED	Blepharotomy / Levator + Muller recession	Anterior	6ᵇ	6.4 ± 1 6 ± 1
Looi 2006	Prospective	99/161	82 (83)	47 ± NA	TED	Levator + Muller recession	Posterior	6-139	7.3 ± 1.4*
Khatavi 2017	Prospective	16/16	12 (75)	45.1 ± 8.8	TED	Levator aponeurectomy	Posterior	6 ± NA	7.8 ± 1.4
Mourits 1999	Prospective	50/78	47 (94)	NA	TED	Levator recession	Anterior	3-9	6.3 ± 1.4*
Zhou 2025	Retrospective	NA/105 NA/71 NA/57 NA/31 Tot 212/264	167 (79)	28.6 ± 8.9	Iatrogenic	Scar lysis / Levator + Muller recession / Levator recession + partial myotomy / Autologous spacer (Orbital fat flap)	Anterior	19.4 ± 4.6	4.1 ± 0.2^†^ 4.9 ± 0.4^†^ 6.3 ± 0.4^†^ 7.3 ± 0.2^†^
Ben Simon 2005	Retrospective	NA/97 NA/61	78 (81)	48 ± 11	TED	Levator + Muller recession + Orbital Decompression/ Levator + Muller recession after Orbital Decompression	Posterior	15 ± 12	5.9 ± 2.1 5.9 ± 1.9
Ben Simon 2005	Retrospective	78/107	63 (81)	49 ± 13	TED	Levator + Muller recession	Posterior	6-84	6 ± 1.9
Ueland 2024	RCT	15/24 15/25	25 (83)	51.5ᵇ	TED	Blepharotomy / Levator recession with adjustable sutures	Anterior	6-18	6.5 ± 0.8^*,^*^§^*
Sun 2018	Prospective	26/32	21 (81)	37.8 ± NA	TED	Non-autologous spacer (Bovine Dermal)	Anterior	6 ± NA	7.7 ± 0.9
Ceisler 1995	Retrospective	37/72	NA	NA	TED	Levator recession + Mullerotomy + Autologous spacer (Muller muscle)	Anterior	24ᶜ	5.5 ± 0.4*
Cho 2012	Retrospective	60/71	57 (95)	34.8 ± NA	Iatrogenic	Autologous spacer (pre-tarsal tissue flap)	Anterior	1-72	5.6 ± 0.5*
Schwarz 2008	Retrospective	15/22	10 (67)	52 ± 15	Mixed	Autologous spacer (deep temporal fascia)	Anterior	12-53	5.1 ± 0.7*
Dixon 1982	Retrospective	12/22	11 (92)	49.3 ± 12.8	TED	Levator recession + Mullerectomy	Anterior	21ᶜ	8.2 ± 1.7*
Doxanas 1981	Retrospective	11/18	NA	NA	Mixed	Homologous spacer (sclera)	Anterior	4-6	6.4 ± 1
Evans 2017	Retrospective	NA/28 NA/66 NA/27	64 (86)	51 ± NA	TED	SO Levator recession + Mullerectomy / SP Levator recession +Mullerectomy / SP Blepharotomy	Anterior	24.2 ± NA	6.7 ± 0.4* 7 ± 0.5* 6.6 ± 0.4*
Hintschich 2005	Prospective	41/60	38 (93)	55 ± NA	TED	Blepharotomy	Anterior	2-36	5.5 ± 1.5*
Demirci 2007	Retrospective	18/21	14 (78)	62 ± 13	Mixed	Blepharotomy	Anterior	13 ± 11	4.2 ± 1
Elner 2003	Prospective	32/50	30 (94)	52 ± 14	TED	Blepharotomy	Anterior	8.5 ± 8.1	6.1 ± 1.3*
Guastella 2022	Retrospective	111/153	93 (84)	52.0 ± 11.9	TED	Blepharotomy + Mullerectomy	Anterior	6ᶜ	10.1 ± 2.1
Hassan 2005	Retrospective	34/34	19 (56)	50 ± NA	Neurologic	Mullerectomy	Posterior	2-66	4.3 ± 1.3
Olver 1995	Retrospective	22/33	19 (86)	49.9 ± NA	TED	Levator division + Mulleretomy	Posterior	6-240 (36ᵇ)	5.8 ± 1.0*
Vu 2022	Prospective	43/46	25 (58)	33.3 ± NA	TED	Autologous Spacer (Orbital septum flap)	Anterior	12-37	6 ± 0.9
Lee JH 2015	Retrospective	22/31 22/26	35 (80)	NA	Mixed	Levator recession + autologous spacer (preaponeurotic fat) / Levator recession	Anterior	20.2 ± 24.8 33.5 ± 34.4	5.9 ± 1.2 6.3 ± 0.9
Lee 2018	Retrospective	19/22	16 (84)	65.1 ± NA	TED	Levator + Muller recession	Posterior	20.9 ± 20.9	6.7 ± 1.3
Soares 1991	Retrospective	10/15	NA	29.7 ± 11.3	TED	Levator + Muller Z-plasty	Anterior	4-60	9.1 ± 2.6*
Ueland 2014	Retrospective	80/86	74 (93)	49ᵇ	TED	Levator recession with adjustable sutures	Anterior	6-123 (56ᵇ)	4.5 ± 0.9*
Mourits 1991	Retrospective	47/78	38 (81)	52 ± NA	TED	Homologous Spacer (sclera)	Anterior	6-42	7.9 ± 2.3*
McNab 2004	Retrospective	21/38	18 (86)	44.3 ± NA	TED	Levator recession with hang-back sutures	Anterior	3ᵃ	8.2 ± 1.3*
Nimitwongsakul 2013	Retrospective	53/78	45 (85)	53.47 ± 12	TED	Blepharotomy	Anterior	15.2 ± 13	NA
Lee J 2015	Retrospective	18/22	12 (67)	53.47 ± 12	TED	Blepharotomy	Anterior	15.2 ± 6	5.2 ± 0.9
Lee JKS 2019	Retrospective	55/77	41 (75)	45.9 ± 11.7	TED	Blepharotomy	Anterior	6 ± 0	7.1 ± 0.7
Pinas 2020	Retrospective	305/471	275 (90)	48.5 ± 12.1	TED	Levator recession +Mullerectomy	Anterior	2-3	7.7 ± 1.1
Putterman 1981	Retrospective	NA/102	NA	NA	TED	Levator recession +Mullerectomy	Posterior	2-72	8.2 ± 1.4
Sales Sanz 2023	Retrospective	52/86	49 (94)	51 ± 10.4	TED	Mullerotomy + graded Müller muscle disinsertion	Anterior	14.4 ± 7.4	6.7 ± 1.1
Short 2011	Retrospective	32/52	32 (100)	53 ± NA	TED	Levator/Müller recession ± Blepharotomy	Anterior	1-12	7.0 ± 1.4
Small 1995	Retrospective	100/181	NA	NA	Mixed	Pre-Whitnall Levator recession/ Levator aponeurosis recession/ Mullerotomy / Mullerectomy	Anterior / Anterior & Posterior / Posterior / Posterior	24ᶜ	NA
Watanabe 2013	Retrospective	10/12	8 (80)	47.5 ± 13	TED	Autologous Spacer (orbital septal flap)	Anterior	6-20	6.3 ± 1.4*
Woog 1996	Retrospective	10/14	10 (100)	44 ± NA	TED	Levator recession with adjustable sutures	Anterior	8.1± 3	6.9 ± 1.7

ᵃminimum; ᵇmedian; ᶜmaximum *Mean or SD estimated from reported range using the method of Hozo et al., BMC Med Res Methodol 2005 or visually estimated from graph or derived from other eyelid retraction measurements (palpebral fissure height, upper eyelid scleral show); §: Preoperative MRD1 values were reported as a single cohort mean (6.5 mm, range 5–8 mm) common to both surgical groups.

SO: septum opening; SP: septum preserving; †Mean and SD for surgical subgroups estimated from severity-based data reported by Zhou et al. (2025); values are approximate

### Change in MRD1 (ΔMRD1)

Across 40 cohorts (2400 eyelids), pooled ΔMRD1 was 2.71 mm (95% CI 2.36-3.06; I²=99.4%) (Fig. 2). Leave-one-out analysis confirmed stability (Fig. 3). Technique-specific effects showed substantial variation (Table 2).

**Fig. 2:**
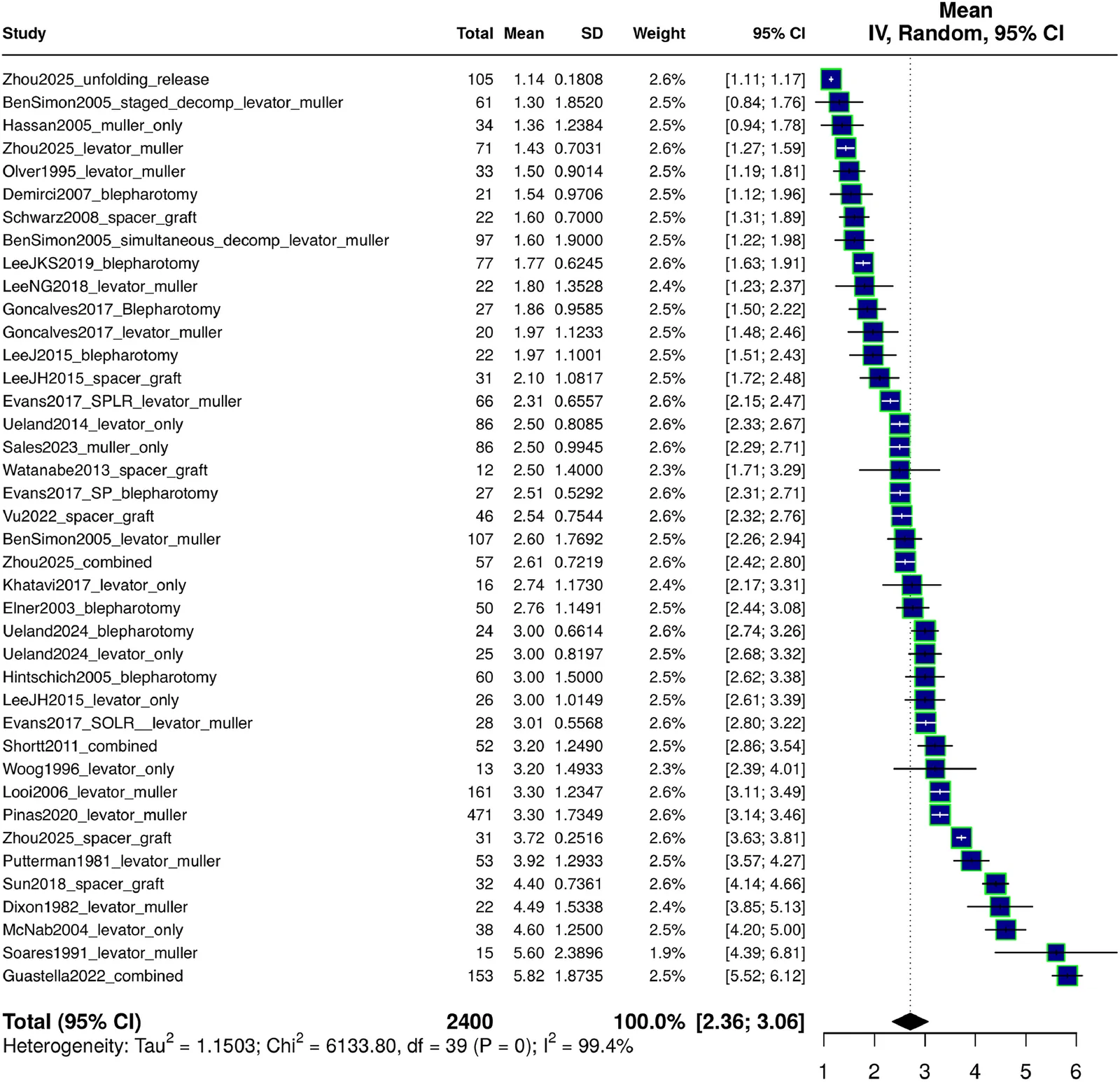
Forest plot of postoperative eyelid lowering (ΔMRD1). Across 40 cohorts (2,400 eyelids), the pooled mean reduction was 2.71 mm (95% CI 2.36-3.06), with very high heterogeneity (τ² = 1.15; I² = 99.4%).

**Fig. 3:**
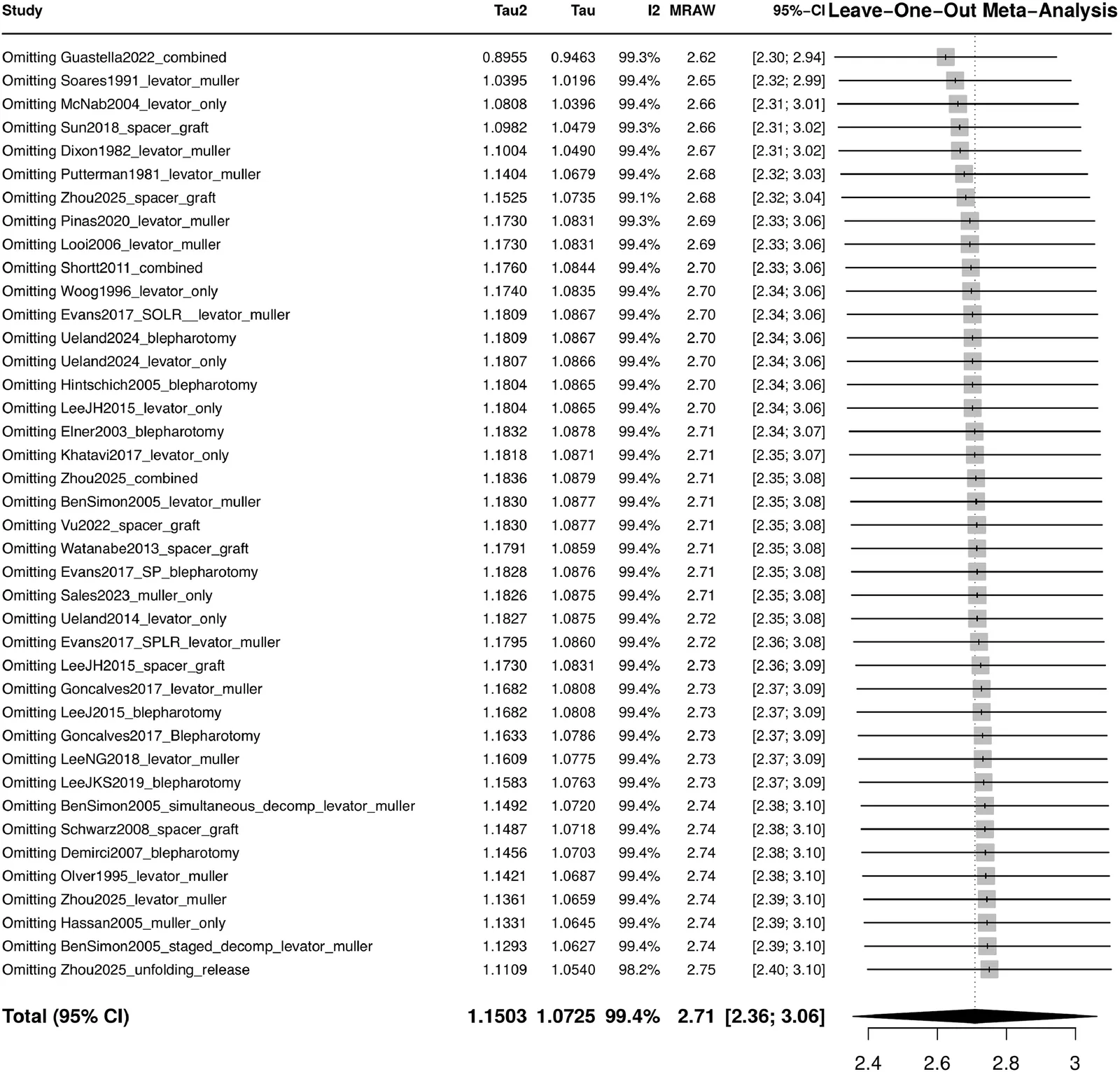
Leave-one-out sensitivity analysis for ΔMRD1. Sequential omission of individual studies confirmed the stability and robustness of the pooled estimate, with values ranging from 2.62 to 2.75 mm.

**Table 2:** ΔMRD1 by surgical technique.

Technique	k	n eyelids	Pooled ΔMRD1 (mm)	95% CI	I² (%)	95% PI	p-value
Blepharotomy	8	308	2.31	1.82-2.79	94.3	0.90-3.71	< 0.0001
Levator/Müller R-R	14	1227	2.68	1.96-3.40	97.8	0.03-5.33	< 0.0001
Levator-only	6	204	3.17	2.38-3.96	94.6	1.13-5.21	< 0.0001
Spacer graft	6	174	2.82	1.71-3.93	98.6	-0.10-5.74	< 0.0001
Combined/Hybrid	3	262	3.88	-0.37-8.12	99.4	-4.59-12.34	< 0.0001
Müller-only	2	120	1.9	-5.3-9.2	95.6	-10.4-14.3	< 0.0001

R-R = recession-resection; PI = prediction interval; I² = heterogeneity index

Multivariable meta-regression explained 76% of heterogeneity. Preoperative severity was the only significant moderator (β=0.72; p<0.0001): each 1-mm increase in baseline retraction predicted 0.72 mm additional lowering (Fig. 4). After severity adjustment, no surgical technique showed independent associations with ΔMRD1 (all p>0.25). In TED (29 cohorts), pooled ΔMRD1 was 2.91 mm (95% CI 2.47-3.34).

**Fig. 4:**
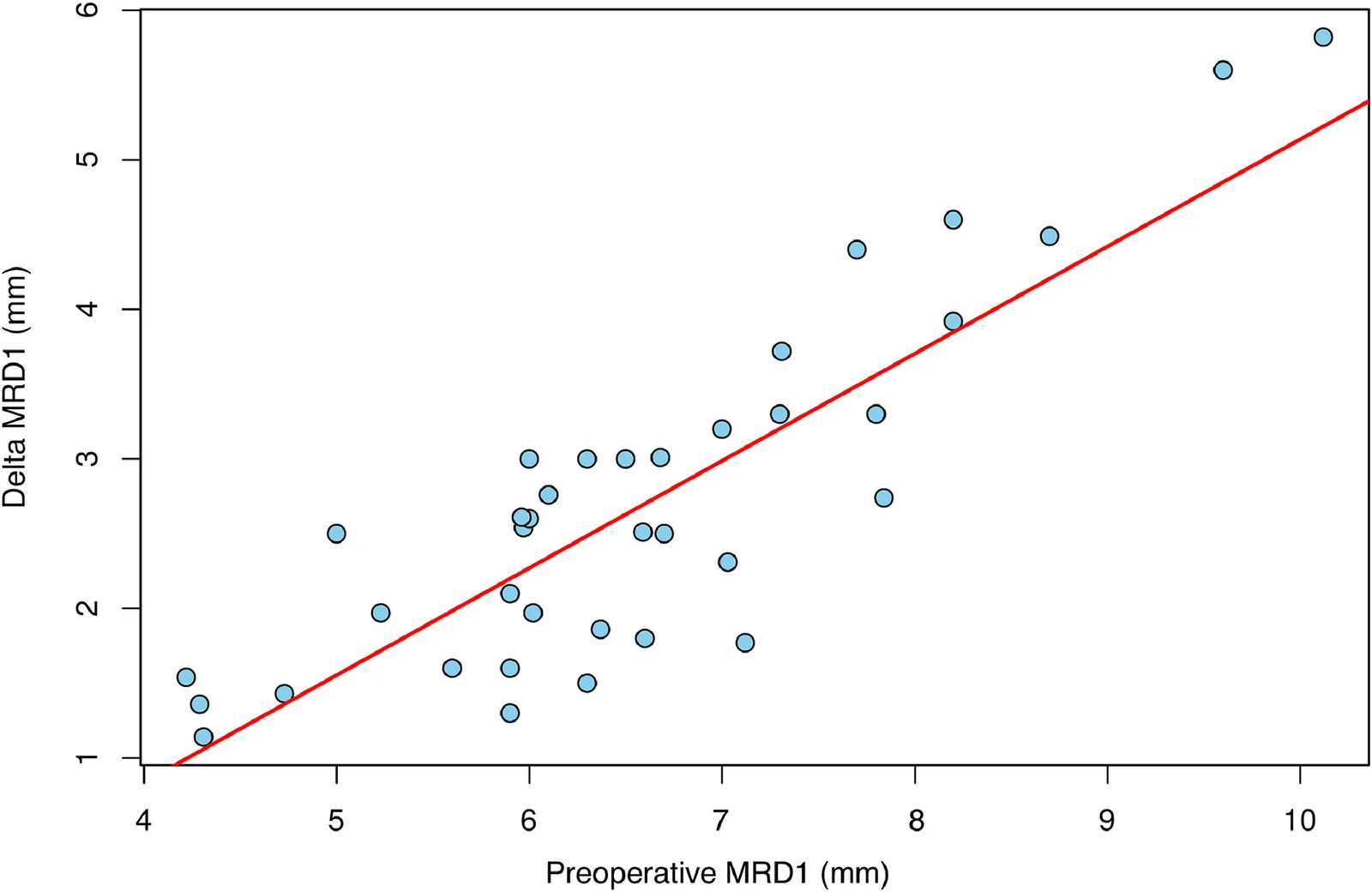
Meta-regression bubble plot showing the relationship between preoperative MRD1 (baseline severity) and postoperative eyelid lowering (ΔMRD1). Each bubble represents a study cohort, with bubble size proportional to study weight. Preoperative severity was the dominant predictor of ΔMRD1 (β = 0.72 mm/mm; p < 0.0001), explaining 75.7% of heterogeneity.

### Surgical success

Across 42 cohorts (2577 eyelids), 85% achieved successful postoperative lid position (95% CI 80.6-89.1%; I²=83.0%) (Fig. 5). No publication bias was detected (Egger t=1.03, p=0.31) (Supplemental Fig. 2). Success rates remained consistently high across all macro-areas (Table 3).

**Fig. 5:**
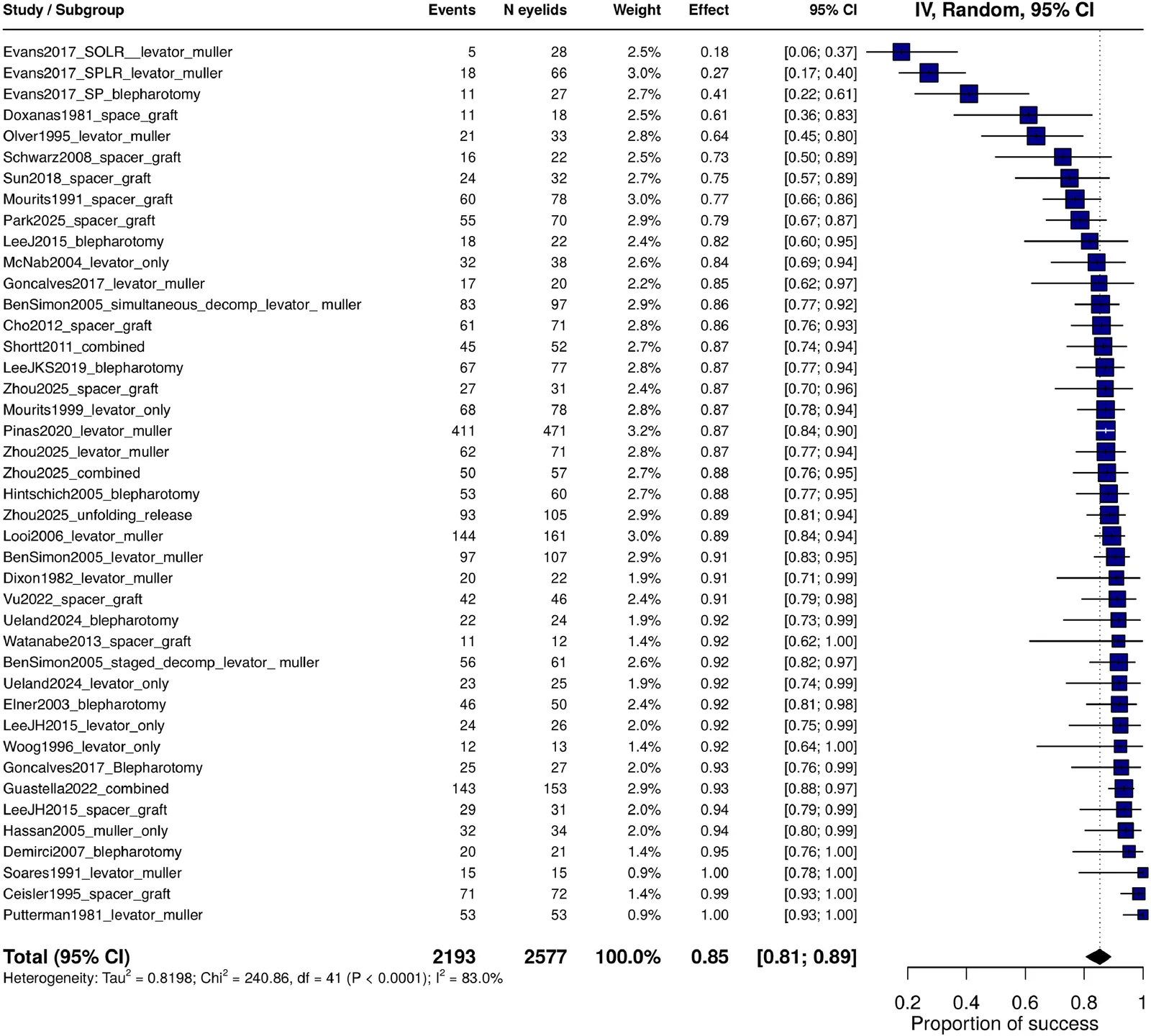
Forest plot of surgical success rates. Across 42 cohorts (2,577 eyelids), the pooled success rate was 85% (95% CI 81-89%), with high heterogeneity (τ² = 0.82; I² = 83.0%).

**Table 3:** Surgical success rates by technique.

Technique*	k	Events/Total	Pooled %	95% CI	I²	95% PI	p-value
Blepharotomy	8	262/308	85.8	71.3-93.6	79.8%	39.8-98.2	< 0.0001
Levator-only	5	159/180	87.9	82.9-91.7	0%	79.3-93.3	0.8077
Levator/Müller R-R	13	1002/1205	82.4	65.0-92.2	92.5%	19.1-98.9	< 0.0001
Spacer graft	11	407/483	82.6	73.5-89.1	57.1%	62.8-93.1	0.0097
Combined/Hybrid	3	238/262	90.3	75.1-96.6	32.9%	76.2-96.5	0.23

R-R = recession-resection; PI = prediction interval; I² = heterogeneity index *Müller-only technique not evaluated due to insufficient data (k=1, n= 34), Unfolding release technique by Zhou not evaluated.

Meta-regression showed neither preoperative severity (p=0.842) nor surgical macro-area predicted success probability, explaining only 11.7% of heterogeneity. TED-related success was 84% (95% CI 76.8-89.5%); iatrogenic retraction showed similar rates at 86% (95% CI 76.9-91.3%).

### Reoperation rates

Across 54 cohorts (3046 eyelids), 12% required reoperation (95% CI 9.1-15.5%; I²=74.2%) (Fig. 6). Funnel plot asymmetry confirmed publication bias (Egger t= -4.42, p<0.0001) (Supplemental Fig. 3). Technique-specific rates showed substantial variation (Table 4).

**Fig. 6:**
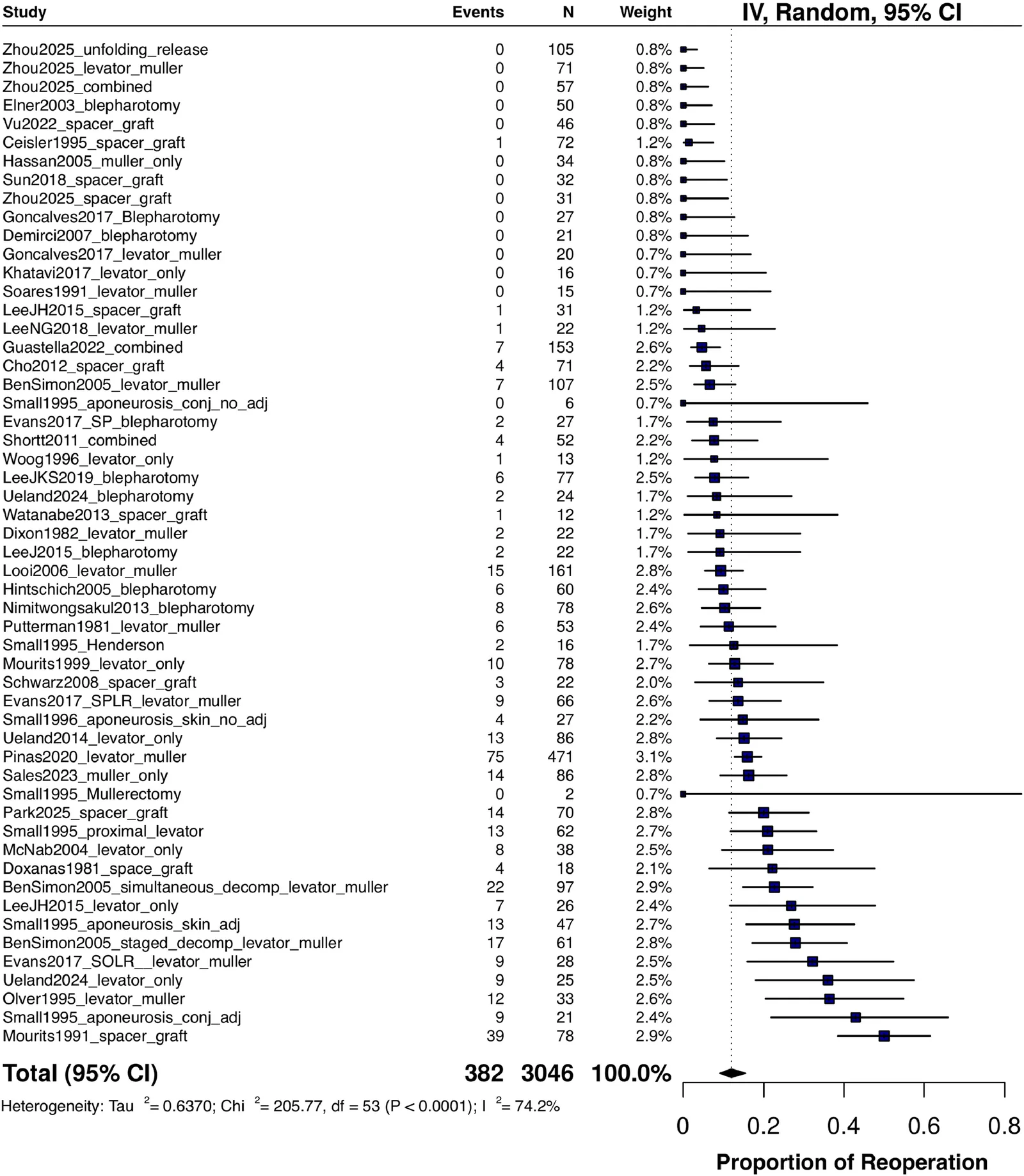
Forest plot of reoperation rates. Across 54 cohorts (3,046 eyelids), the pooled reoperation rate was 11.9% (95% CI 9.1-15.5%), with substantial heterogeneity (τ² = 0.64; I² = 74.2%).

**Table 4:** Reoperation rates by technique.

Technique*	k	Events/Total	Pooled %	95% CI	I²	95% PI	p-value
Blepharotomy	9	26/386	8.2	5.8-11.6	0%	5.37-12.46	0.73
Levator-only	12	87/445	21.3	15.4-28.7	45.6%	11-38	0.042
Levator/Müller R-R	15	177/1243	14.4	9.2-21.7	70.9%	4-40	< 0.0001
Spacer graft	11	67/483	8.6	3.3-20.5	85.4%	1-63	< 0.0001
Combined/Hybrid	3	11/262	5.1	1.3-18.5	18.1%	1.43-16.48	0.30
Müller-only	3	14/122	14.96	3.7-44.8	37.4%	4.9-37.1	0.202

R-R = recession-resection; PI = prediction interval; I² = heterogeneity index * Unfolding release technique by Zhou not evaluated

Multivariable meta-regression explained 70% of heterogeneity. Preoperative severity showed no association (p=0.63). Two techniques demonstrated significantly elevated risk: levator-only (OR=3.5; p=0.014) and levator/Müller recession-resection (OR=2.5; p=0.034). TED reoperation was 13% (95% CI 9.8-17.4%); iatrogenic showed 12% (95% CI 6.1-20.9%).

### Overcorrection and undercorrection

Across 49 cohorts (2864 eyelids), overcorrection occurred in 6% (95% CI 4.2-7.5%; I²=51.6%) (Fig. 7) and undercorrection in 7% (95% CI 5.3-9.1%; I²=60.6%) (Supplemental Fig. 4). Subgroup analyses showed low rates across macro-areas (Supplemental Tables 3, 4).

**Fig. 7:**
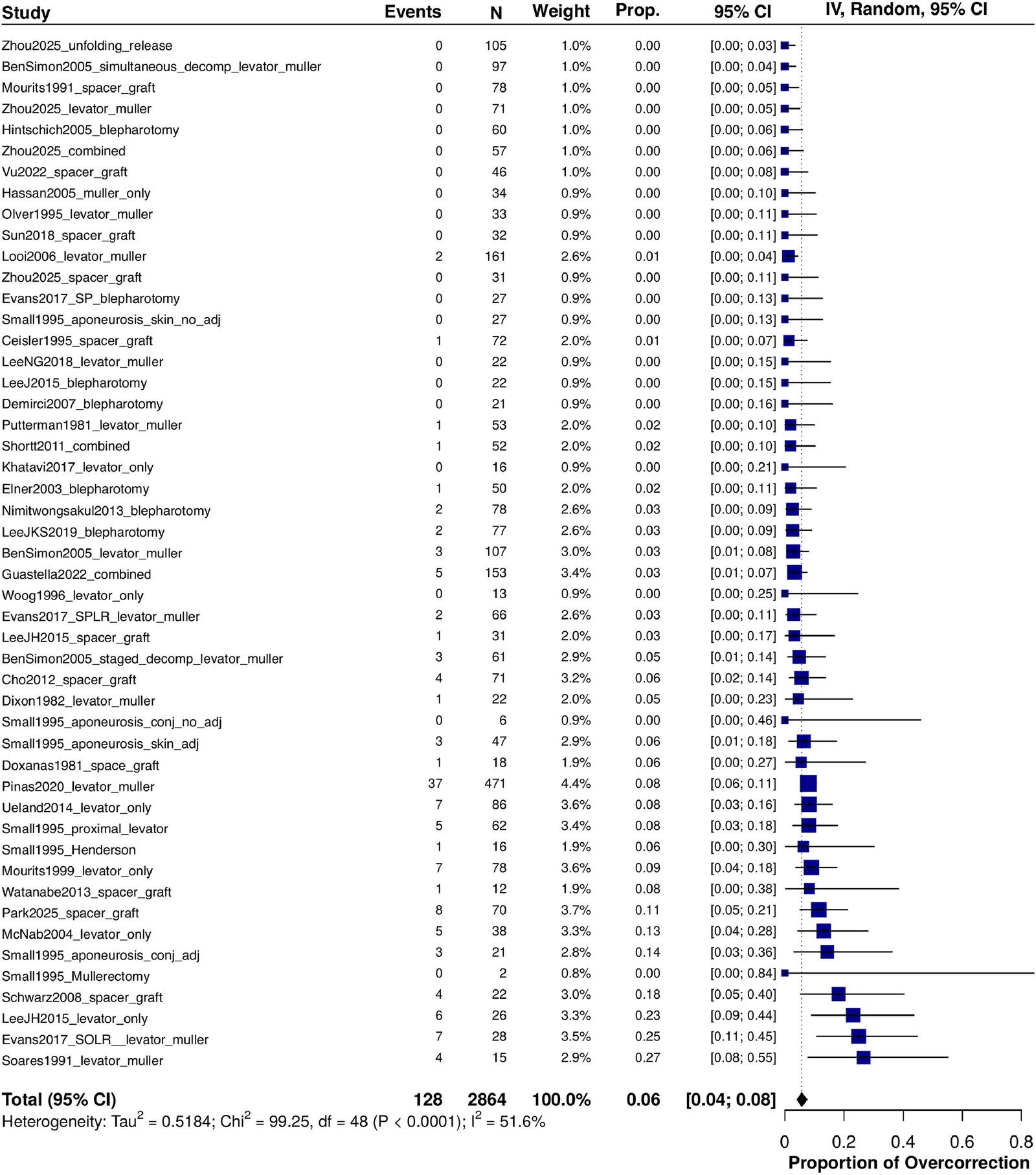
Forest plot of overcorrection rates. Across 49 cohorts (2,864 eyelids), the pooled overcorrection rate was 5.7% (95% CI 4.2-7.5%), with moderate heterogeneity (τ² = 0.52; I² = 51.6%).

Meta-regression revealed distinct predictive patterns. Overcorrection was unrelated to preoperative severity (p=0.157) but technique-dependent: levator-only procedures showed significantly elevated risk (OR=5.6; p=0.020). Conversely, undercorrection was inversely related to preoperative severity (β=−0.33; p=0.045), with no significant technique associations. These findings indicate undercorrection is severity-dependent, whereas overcorrection is technique-dependent.

### Lagophthalmos/keratopathy resolution

Across 14 cohorts (358 eyelids), pooled resolution was 72% (95% CI 54.5-84.6%; I²=78.9%) (Supplemental Fig. 5). Anterior approaches achieved 78% (95% CI 63.0-88.4%) versus 44% (95% CI 3.9-93.7%) for posterior approaches (p=0.044). However, the posterior subgroup (k=3) demonstrated marked instability on leave-one-out analysis, reflecting limited evidence.

### Eyelid cosmetic abnormalities

Cosmetic abnormalities were reported in 12% (95% CI 9.1-16.3%; I²=76.5%), but 81% of cohorts reported zero events, compromising estimate reliability. Meta-regression found no significant predictors. Rates ranged from 1% (blepharotomy) to 9% (levator-only), though confidence intervals overlapped substantially.

### Spacer materials analysis

Across 11 spacer-graft cohorts (483 eyelids), autologous grafts (7 cohorts; 285 eyelids) achieved 88% success (95% CI 77.9-94.2%) with 5% reoperation (95% CI 2.3-11.5%). Non-autologous materials (4 cohorts; 198 eyelids) yielded 76% success (95% CI 65.8-83.2%) with substantially higher reoperation at 25% (95% CI 4.0-72.0%) (Table 5).

**Table 5:** Spacer graft outcomes.

Graft Type	k	n eyelids	Success Rate % (95% CI)	I²	95% PI Success Rate	Reoperation Rate % (95% CI)	I²	95% PI Reoperation
Autologous	7	285	88.3 (77.9-94.2)	46.9%	72-95	5.3 (2.3-11.5)	17.6%	2-12
Non-autologous	4	198	75.6 (65.8-83.2)	0%	64-84	24.7 (4.0-72.0)	86.1%	1-80
Overall	11	483	82.6 (73.5-89.1)	57.1%	63-93	8.6 (3.3-20.5)	85.4%	1-63

PI = prediction interval; I² = heterogeneity index

### Publication bias and risk of bias assessment

Publication bias was detected for reoperation (Egger t=−4.42, p<0.0001) (Supplemental Fig. 3) but not for ΔMRD1, success rates, or over/undercorrection outcomes. Risk of bias was assessed using ROBINS-I for non-randomized studies and RoB 2 for randomized trials. Three studies (8%) achieved low risk of bias; 28 (72%) were moderate risk and eight (21%) serious risk (Supplemental Table 5). The principal sources of bias were confounding by indication and selection bias inherent to single-arm retrospective designs.

## Discussion

The surgical management of upper eyelid retraction (UER) remains technically heterogeneous, with no single procedure emerging as a universally accepted standard. Treatment aims to provide functional relief of lagophthalmos, exposure keratopathy, and achievement of aesthetically acceptable eyelid position.^17^^,^^18^ A consistent organizing principle is identifying whether pathology lies in the anterior (skin, orbicularis), middle (orbital septum and fat), or posterior lamella (Levator/Müller complex, conjunctiva). Posterior lamellar surgical strategies include *transconjunctival approaches* (Müller recession with or without levator disinsertion),^7^^,^^10^^,^^18^, ^19^, ^20^, ^21^, ^22^, ^23^, ^24^, ^25^ which avoid cutaneous scarring,^18^ but may exacerbate dry eye^26^; or *anterior (transcutaneous) approaches* (levator recession, graded blepharotomy, adjustable sutures) (Figs. 8, 9)^5^^,^^13^^,^^19^^,^^27^, ^28^, ^29^, ^30^ that include *spacer graft techniques,* with autologous tissues or allogeneic materials, usually reserved for severe cases (Fig. 10).^4^^,^^9^^,^^11^^,^^31^, ^32^, ^33^, ^34^, ^35^, ^36^, ^37^, ^38^ The proliferation of techniques and limited comparative data highlight the lack of evidence-based surgical guidelines. A recent Graves-only scoping review,^16^ did not provide a quantitative synthesis of surgical outcome determinants. This meta-analysis synthesized data from 39 studies (retrospective 77%; prospective 18%, RCTs 5%) with 2064 patients (3096 eyelids). Baseline severity was highly variable (mean MRD1 6.5±1.3 mm, range 4.2-10.1 mm), reflecting that patients presenting for surgery typically exhibit moderate-to-severe retraction with functional and cosmetic impairment.

**Fig. 8:**
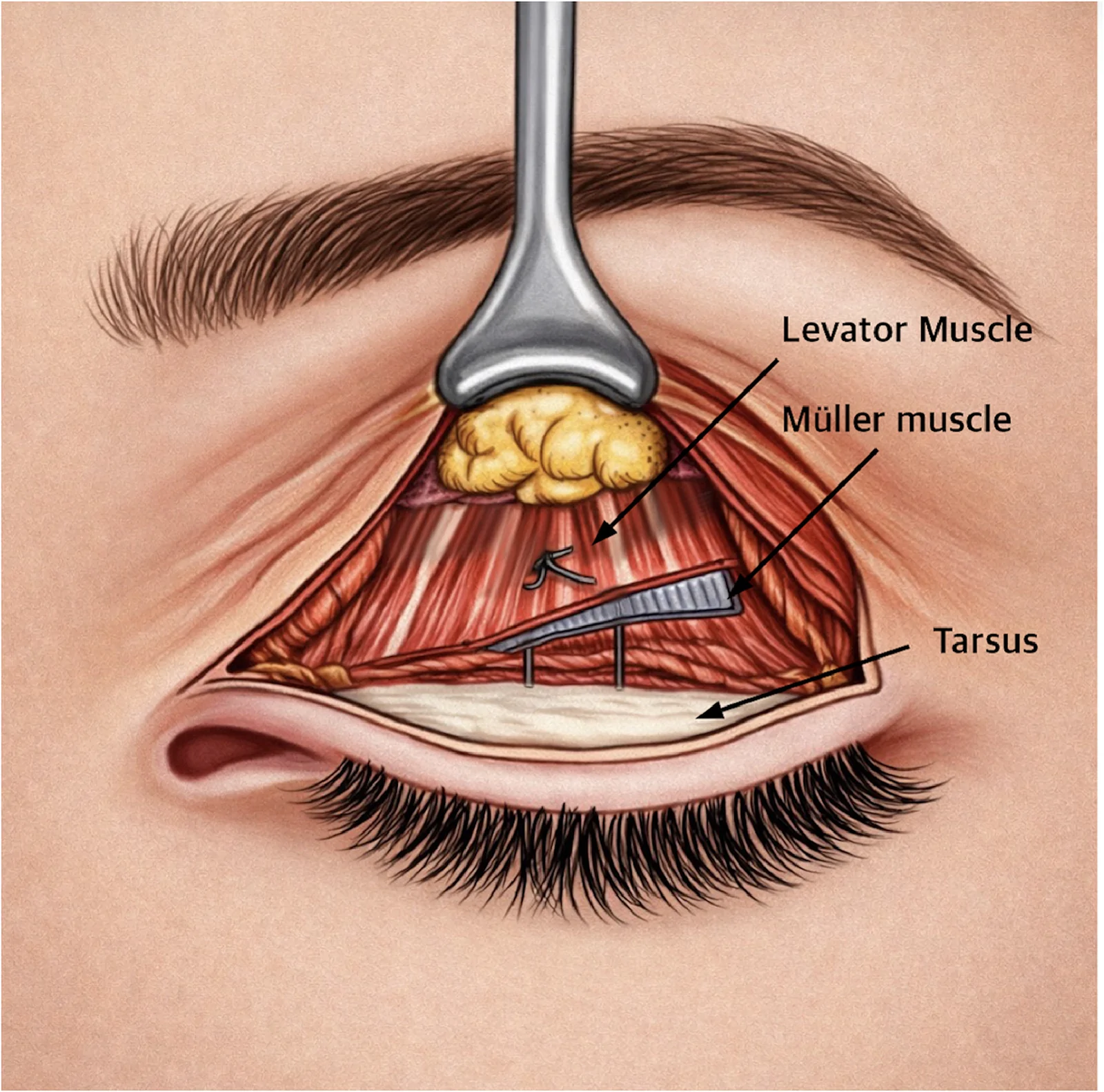
Anterior levator aponeurosis-Müller muscle recession for upper eyelid retraction, illustrating controlled recession of the retractor complex from the tarsus.

**Fig. 9:**
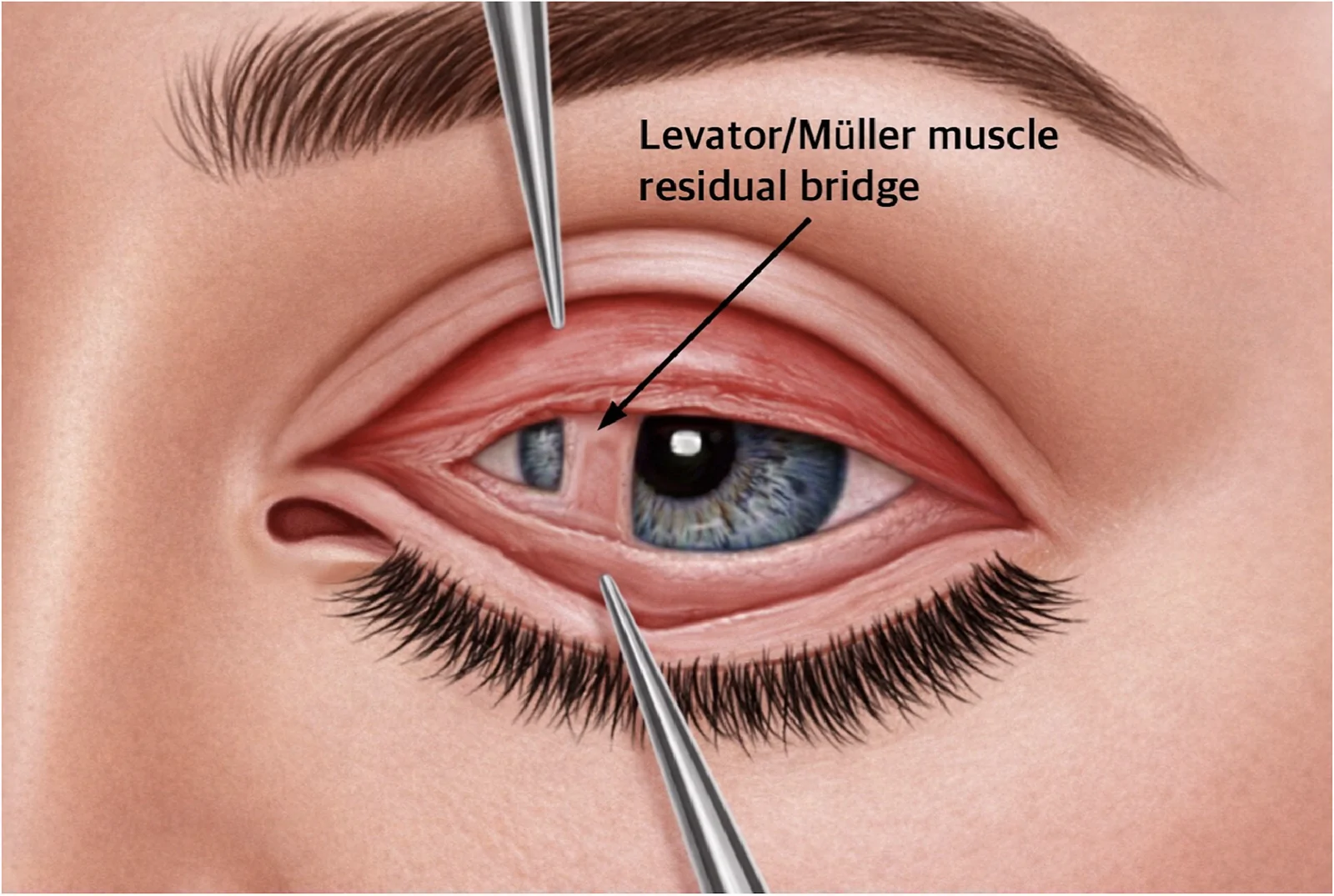
Graded full-thickness anterior blepharotomy (FTAB) for upper eyelid retraction, showing full-thickness transection with preservation of a central levator/Müller muscle bridge to maintain function and contour.

**Fig. 10:**
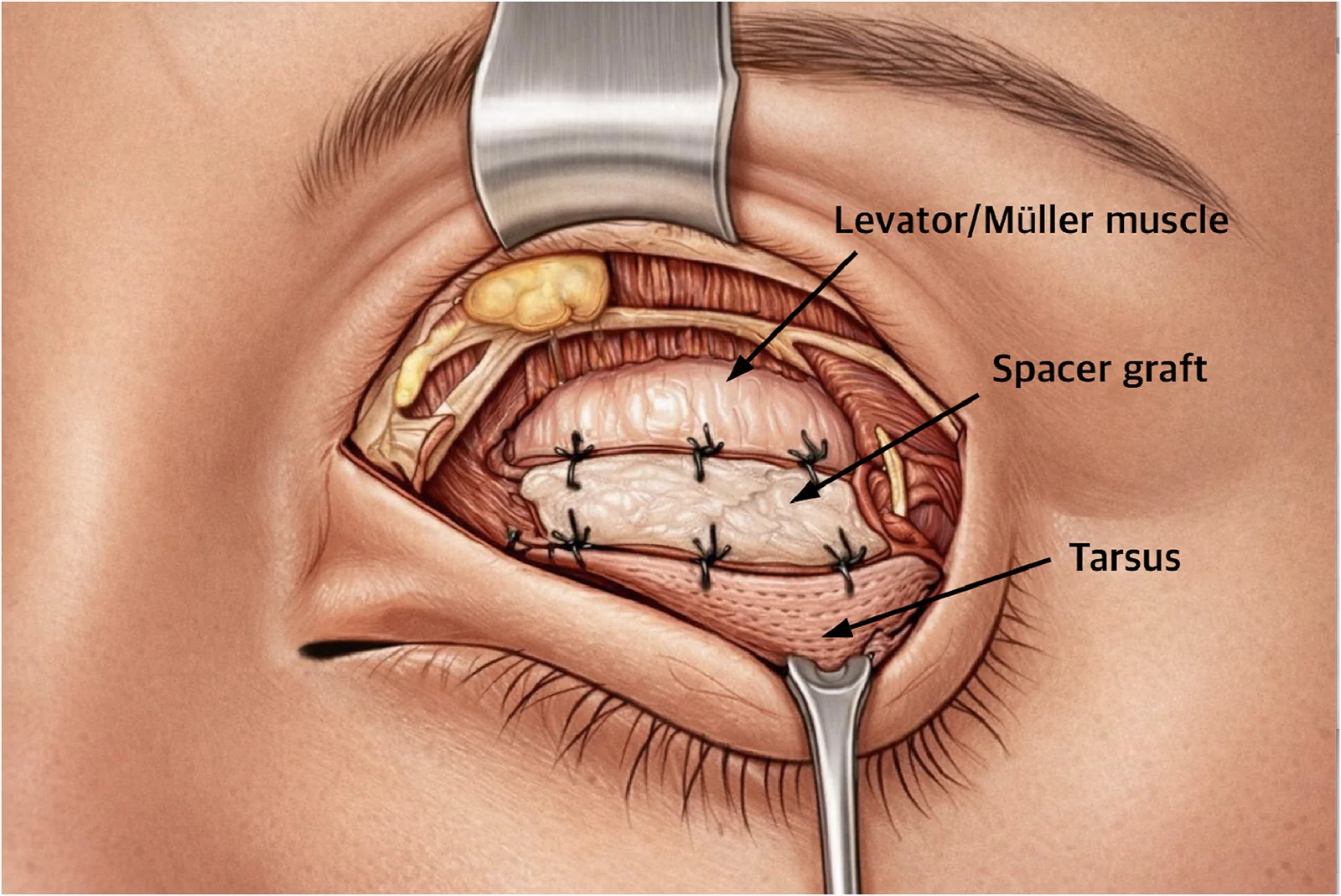
Spacer graft technique for correction of severe or complex upper eyelid retraction, illustrating interposition of a graft between the levator/Müller complex and the tarsus to achieve eyelid lengthening. Autologous and allogeneic materials may be used, whereas banked sclera and non-biologic meshes are now largely abandoned due to higher complication rates.

### Preoperative severity as the primary determinant of eyelid lowering

The amount of eyelid lowering achieved is determined primarily by how retracted the eyelid is before surgery, not by which technique is used. Although the single-arm design precludes direct comparisons among techniques, meta-regression explained 76% of heterogeneity across 40 cohorts, with baseline MRD1 as the only significant moderator (β=0.72 mm per mm; p<0.0001). Once severity was accounted for, no technique showed an independent effect on ΔMRD1 (all p>0.25). Elner et al. demonstrated this directly: severe cases lowered by 4.2 mm, moderate by 2.6 mm, and mild by only 1.4 mm, yet all three groups converged toward a postoperative MRD1 of approximately 3.0–3.5 mm.^27^ Regardless of technique, the surgeon titrates eyelid height intraoperatively until the target MRD1 of approximately 3.0-3.5 mm is achieved.

### Stable success despite variable ΔMRD1

Across 42 cohorts (2,577 eyelids), surgical success clustered around 85% across techniques. Neither preoperative severity nor surgical macro-area significantly predicted success, despite substantial variation in absolute ΔMRD1 across macro-areas (range 2.31-3.88 mm). Technique selection should therefore be judged less by lowering magnitude than by durability, complication profile, and revision risk. To translate these findings into practice, we propose a severity- and complication-based decision algorithm (Fig. 11).

**Fig. 11:**
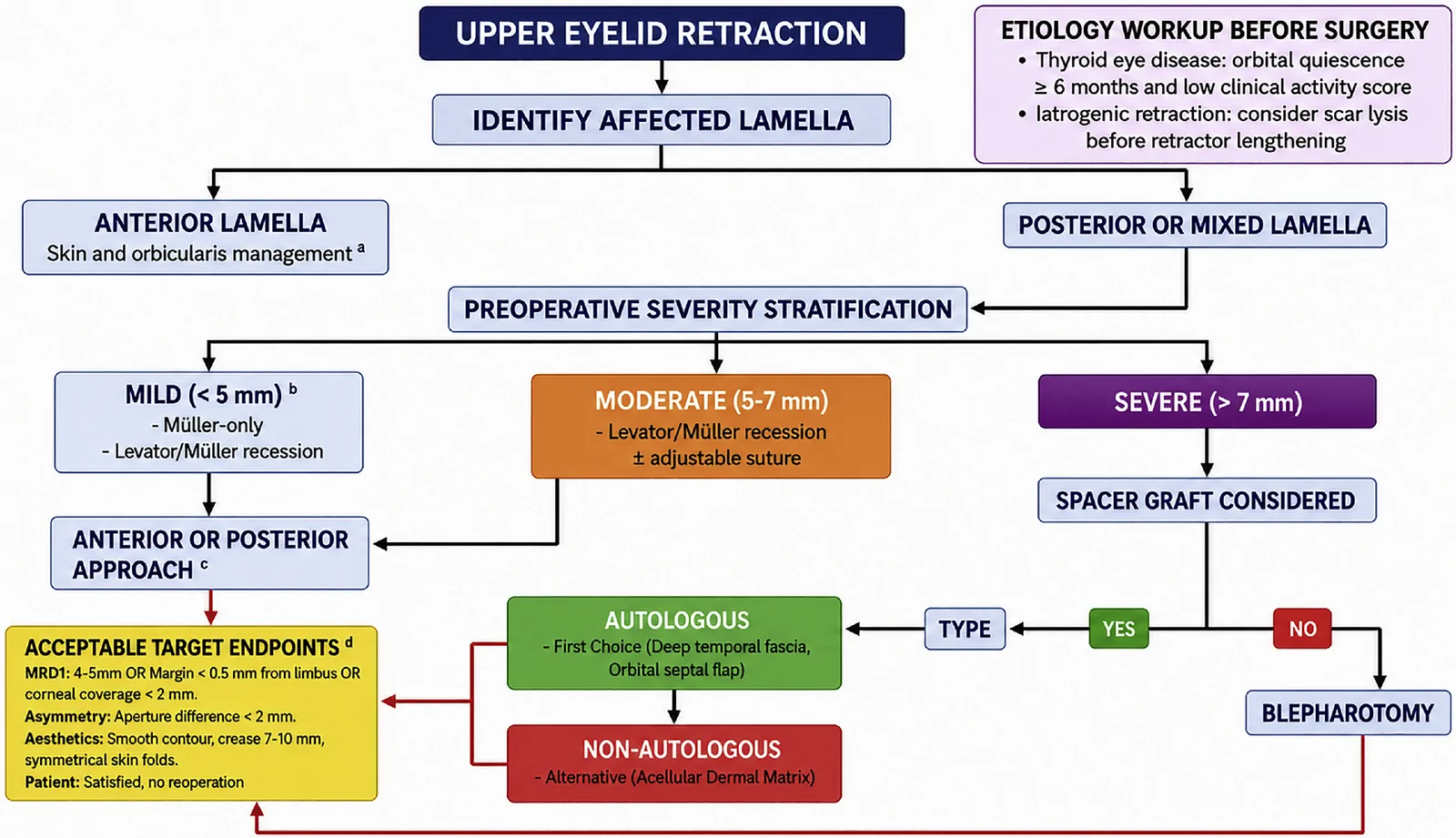
Suggested algorithm for surgical management of upper eyelid retraction. Postoperative lowering power was primarily associated with baseline severity and showed no independent association with surgical technique. Severity tiers represent a pragmatic approach based on literature review and illustrate scenarios in which different techniques may be considered. ^a^*Anterior lamellar procedures are outside the scope of this meta-analysis. ^b^Higher risk of undercorrection* (β=-0.33; p=0.045). ^c^*Anterior approaches achieved higher lagophthalmos/keratopathy resolution (78.3%) compared with posterior approaches (43.6%; p=0.044). ^d^ Based on Perfect and Acceptable Mourits and Sasim criteria.*

### Reoperation risk: the main technique-level differentiator

Technique choice did not independently predict lowering magnitude, but it did predict durability. Reoperation rates were the major differentiator in this analysis: meta-regression explained 70% of heterogeneity, and unlike ΔMRD1, reoperation risk was driven by technique rather than by baseline severity (p=0.63). Blepharotomy showed the lowest and most consistent revision rates (8%; I²≈0%), meaning that across all series, roughly one in twelve operated eyelids required a second procedure. Levator-only procedures, by contrast, carried a 3.5-fold higher reoperation risk (OR=3.5; p=0.014), with a pooled rate of 21%, meaning more than one in five eyelids eventually required revision. Levator/Müller recession–resection fell in between, with a 2.5-fold elevated risk (OR=2.5; p=0.034) and a pooled reoperation rate of 14%, consistent with the 20–30% revision rates reported after adjustable suture techniques in individual series.^13^^,^^29^^,^^30^^,^^39^^,^^40^ A plausible mechanism is that recession techniques preserve conjunctival continuity and leave posterior fibrosis partially intact, maintaining a pathway through which contractile forces can gradually reassert, pulling the eyelid back up over time. Full-thickness blepharotomy, by creating a controlled gap across the entire eyelid thickness, may heal as an endogenous spacer, producing more stable long-term lengthening. Spacer graft outcomes varied substantially depending on material choice, and are discussed separately below. One important caveat is publication bias detected for reoperation rates (Egger t=-4.42; p<0.0001), suggesting that series with higher revision rates are likely underrepresented in the literature. The true reoperation burden across techniques may therefore be somewhat higher than these pooled estimates reflect.

### Overcorrection and undercorrection: divergent risk profiles

Overcorrection and undercorrection followed distinct risk profiles. Overall, overcorrection occurred in 6% of eyelids (95% CI 4.2-7.5%) and undercorrection in 7.0% (95% CI 5.3-9.1%), rates consistent with the 1-10% range reported across individual series.^11^^,^^27^^,^^41^ Meta-regression showed that undercorrection was inversely associated with preoperative severity (β=−0.33; p=0.045), with no independent effect of surgical technique: the more retracted the eyelid before surgery, the less likely it is to be undercorrected. A severely retracted eyelid (MRD1 = 9 mm) has a large margin before the surgeon risks leaving it too high, whereas a mildly retracted eyelid (MRD1 = 5 mm) leaves less margin for error. No surgical technique independently predicted undercorrection, suggesting it reflects severity and case selection more than technical execution.

Overcorrection showed the opposite pattern. It was unrelated to baseline severity (p=0.157) but strongly technique-dependent: levator-only procedures carried a 5.6-fold higher overcorrection risk (OR=5.6; 95% CI 1.35–23.26; p=0.020), with a pooled overcorrection rate of 11%, nearly double the overall average. This pattern is consistent with randomized and cohort data showing that levator recession with adjustable sutures carries a higher rate of postoperative ptosis or excessive lowering.^28^^,^^39^ This supports careful intraoperative titration when performing levator-only recession, because lower baseline MRD1 does not appear to protect against overshooting. Reduced orbicularis tone, whether related to TED myopathy, facial nerve palsy, or post-decompression changes, may further amplify the functional effect of retractor lengthening; however, this variable was not systematically reported and should be addressed in future prospective studies.

### Functional outcomes: lagophthalmos and keratopathy resolution

Lagophthalmos and exposure keratopathy resolution captures the functional success of the operation beyond eyelid position alone. Across 14 cohorts (358 eyelids), pooled resolution was 72% (95% CI 54.5–84.6%). Anterior approaches achieved higher resolution rates than posterior approaches (78% vs 45%; p=0.044), but this difference should be considered carefully since the posterior subgroup included only three studies, leave-one-out analysis showed substantial instability, and the wide confidence interval (95% CI 3.9–93.7%) reflects the unreliability of that estimate. A possible explanation is that full-thickness blepharotomy directly addresses fibrosis across all eyelid layers and may more effectively restore dynamic closure mechanics,^5^^,^^27^ but this remains hypothesis-generating. Posterior approaches have been criticized for lacrimal ductule injury and dry eye exacerbation,^14^^,^^42^ yet the only RCT comparing approaches found no significant difference in dry-eye outcomes,^42^ and multiple posterior series report high patient satisfaction.^18^^,^^21^^,^^24^^,^^43^ Rarer approach-specific complications, including direct corneal abrasion or conjunctival injury from the transconjunctival route, were reported only sporadically across individual series^21^^,^^32^ and occurred too infrequently to support pooled estimates. Their absence from quantitative synthesis should not be interpreted as absence of clinical relevance, but as a limitation of the available data granularity.

### Spacer graft material selection

Across the included literature, spacer interposition emerges as an escalation strategy reserved when retractor recession alone is unlikely to provide adequate lengthening. This applies most consistently in severe TED-related retraction, where marked fibrosis and myopathic shortening of the levator-Müller complex limit the effectiveness of recession alone^31^^,^^32^^,^^35^^,^^37^^,^^43^, ^44^, ^45^; and revision or iatrogenic cases with scarred, non-compliant tissue planes, where a spacer restores a physical scaffold that recession cannot replicate.^9^^,^^11^^,^^44^ Conversely, several series report levator recession alone achieving adequate correction across a broad severity range without spacer placement,^17^^,^^21^ indicating that spacer use, while frequently favored in severe or revision cases, is not universally mandatory. When a spacer is indicated, autologous tissue appears preferable. Autologous grafts (deep temporal fascia, orbital septal or fat flaps) achieved 88% success (I²=46.9%) with 5% reoperation and low heterogeneity (I²=18%), meaning this performance was consistent across series. When autologous grafts did require revision, it was typically for fine height adjustment rather than graft failure.^9^^,^^11^^,^^35^^,^^37^^,^^38^^,^^44^^,^^46^ Within the autologous subgroup, six of seven cohorts used vascularized tissue, either as a transposition flap (orbital septal/fat flap^9^^,^^35^^,^^37^; preaponeurotic fat advancement^38^; pretarsal tissue flap^44^) or as a pedicled in situ spacer (Müller muscle)^46^; only one study was pooled as non-vascularized free graft (deep temporal fascia^11^). Additional non-vascularized options, including dermis-fat graft,^47^ fascia lata,^48^ free tarsal graft^49^ and labial mucosa^50^ have been described in case reports but did not meet the inclusion criteria of the present meta-analysis. Given the marked imbalance between subgroups (6 vs 1 cohort), a statistically meaningful comparison of vascularized versus non-vascularized autologous spacers was not feasible. Non-autologous materials achieved 76% success and 25% reoperation, a nearly five-fold higher revision rate, with high heterogeneity (I²=86%) reflecting unpredictable performance across series. Acellular dermal matrices and donor sclera showed variable absorption and foreign-body reactions,^4^^,^^31^^,^^32^^,^^45^ with AlloDerm® (LifeCell Corporation, Bridgewater, New Jersey, USA) reported to undergo greater than 50% resorption at six months. Synthetic mesh materials, despite isolated favorable reports, carried unacceptably high extrusion rates.^34^ The tradeoff for autologous graft as donor-site morbidity and technical complexity, is justified by substantially more reliable integration and a fourfold reduction in reoperation risk compared to available alternatives.

### Revision surgery strategies

No pooled data were retrievable to analyze revision strategy after failed primary surgery. However it appears to be technique- and surgeon-dependent rather than standardized. Some authors reserve full-thickness blepharotomy for recurrence after failed posterior Müller muscle recession, arguing it can divide residual fibrotic adhesions in a single step,^18^ while others propose the opposite sequence, converting a failed blepharotomy to levator recession with adjustable sutures to allow fine intraoperative titration in unpredictable scarred tissue.^13^ In parallel, spacer grafts are favored in scarred or iatrogenic revision beds, where dense fibrosis limits accurate isolation of the retractors and a spacer provides mechanical scaffolding that recession alone cannot replicate.^9^^,^^11^^,^^44^ This divergence indicates that revision technique selection currently rests on individual surgeon experience rather than comparative evidence and should be investigated in future studies. Local corticosteroid injection has been described as a non-surgical treatment for active-phase TED-related retraction, where it may reduce inflammatory-driven Müller muscle stimulation. However, none of the included studies evaluated steroid injection as a prophylactic measure to prevent surgical recurrence or postoperative scar contracture, a distinction that lies outside the scope of the present surgical outcome data and warrants dedicated investigation.

### Etiology-specific considerations

Despite their different origins (inflammatory fibrosis in TED, mechanical overcorrection in iatrogenic cases, loss of orbicularis tone in paralytic retraction), upper eyelid retraction from different etiologies converges on the same final pathway: excess tension within the levator–Müller complex. This shared final pathway may partly explain why surgical outcomes appeared similar across etiologies: success rates of 84% versus 86%, and reoperation rates of 13% versus 12%, for TED and iatrogenic cohorts respectively.^5^, ^6^, ^7^^,^^9^^,^^37^^,^^44^ Etiology alone should not drive technique selection; however, several TED-specific considerations deserve attention. Upper eyelid retraction in TED reflects several interrelated mechanisms in addition to the passive effect of globe prominence. During the active phase, adrenergic stimulation of Müller muscle may contribute a reversible component of eyelid elevation; with disease progression, inflammatory infiltration and fibrotic contracture of the levator-Müller complex may fix the eyelid in a retracted position; and restrictive extraocular myopathy, particularly inferior rectus involvement, may influence eyelid position through altered fixation effort and levator recruitment.^14^ These mechanisms support the standard rehabilitative principle that eyelid surgery should generally be deferred until TED is inactive and eyelid position is stable. In the included literature, when disease activity was reported, surgery was performed in the quiescent or stable phase. The role of orbital decompression should be interpreted separately from that of eyelid-lowering surgery. Decompression is essential when indicated for proptosis or compressive optic neuropathy, but it addresses orbital expansion and globe prominence rather than the eyelid retractors themselves. In the TED cohorts included in the present meta-analysis, direct eyelid surgery produced a pooled MRD1 reduction of 2.91 mm (95% CI 2.47-3.34). A previous meta-analysis reported that orbital decompression alone reduces MRD1 by only 0.35 mm on average,^51^ suggesting a statistically measurable but clinically limited effect on upper eyelid position. Therefore, decompression should be considered complementary to, rather than a substitute for, eyelid-lowering surgery when residual upper eyelid retraction persists, and is essential when indicated for proptosis, exposure, or compressive optic neuropathy. Selected studies have reported comparable eyelid outcomes after staged versus combined decompression and eyelid surgery, and both treatment contexts were represented in the included literature.^43^^,^^52^ Although exophthalmometry was measured or discussed in several TED series, reporting was too heterogeneous to harmonize as a covariate for technique-specific analyses. Some studies reported individual Hertel values, others provided group-level summaries or severity categories, and some excluded significant proptosis or enrolled patients after decompression.^11^^,^^31^ As a result, proptosis and decompression status could not be reliably incorporated as moderators in the present meta-analysis. Future head-to-head comparative studies may clarify the role of eyelid vs decompression surgery in TED. Regarding the iatrogenic cluster, none of the included studies used skin grafting as the primary corrective strategy; all targeted the levator-Müller complex directly.^9^ This supports a retractor-lengthening approach as the default for UER correction across etiologies, reserving anterior lamellar reconstruction only for cases with clear skin or orbicularis insufficiency.

### Cosmetic outcomes: an underreported domain

Cosmetic outcomes remain the most poorly documented domain in retraction surgery.^22^ In this analysis, 81% of cohorts reported zero aesthetic abnormalities, a figure that almost certainly reflects underreporting rather than true absence of complications. When cosmetic outcomes were described, they included contour irregularities, crease abnormalities, and disproportionate tarsal platform show, with rates ranging from 1% after blepharotomy to 9% after levator-only procedures, though overlapping confidence intervals precluded meaningful comparisons. This reporting gap is increasingly difficult to justify. A successful MRD1 does not equal a satisfactory eyelid: a lid can sit at 3.5 mm and still look wrong to the patient. Recent work has highlighted the relationship between MRD1 change and tarsal platform elongation,^24^ and image-based tools, including Bézier-curve contour analysis and automated asymmetry indices, have been proposed to capture subtle contour abnormalities that ruler measurements miss.^20^^,^^53^ Structured outcome frameworks integrating height, contour, symmetry, and patient-reported satisfaction exist^17^ but remain inconsistently applied across studies. Future trials should treat aesthetic outcomes as co-primary endpoints alongside MRD1, adopting standardized metrics and validated patient-reported questionnaires.

### Limitations

The evidence base for retraction surgery reflects the inherent challenges of studying a technically demanding, low-volume procedure. Most included studies were retrospective single-surgeon series, and only two randomized controlled trials were available. Confounding by indication was pervasive, though meta-regression accounting for preoperative MRD1 partially addressed this limitation. Publication bias was detected for reoperation rates, suggesting true revision burden may be somewhat higher than pooled estimates reflect. Complication analyses were constrained by data availability; rarer events (graft reactions, corneal injury, granulomas) were too inconsistently reported to support reliable pooled estimates. Anesthesia type, intraoperative titration strategy, surgeon experience, and learning-curve effects may influence outcomes and limit generalizability. Primary and revision cases were not consistently distinguished, despite their different tissue planes, compliance, and capacity for graded intraoperative adjustment. Future studies should stratify outcomes by primary versus revision status, as pooling these populations likely inflates heterogeneity and dilutes technique-specific signals. Finally, horizontal eyelid laxity was rarely captured, although it may modify both final MRD1 and contour; concurrent canthal tightening should therefore be considered when clinically indicated rather than assumed unnecessary.

## Conclusions

This meta-analysis of 39 studies and more than 2000 patients shows that surgical correction of upper eyelid retraction achieves approximately 85% success across techniques when matched to disease severity. Preoperative MRD1, rather than a single technique, is the dominant determinant of eyelid lowering outcome. Reoperation risk should be the primary consideration in surgical planning and preoperative counselling. Surgeons managing upper eyelid retraction should be proficient across the procedural spectrum and tailor their approach to the individual patient's anatomy, severity, and surgical history.

## Declaration of AI and AI-assisted technologies in the writing process

During the preparation of this work, the authors used ChatGPT (OpenAI) and Claude (Anthropic) in order to improve readability and language editing. After using these tools, the authors reviewed and edited the content as needed and take full responsibility for the content of the publication.

## Disclosures

The authors report no relationships, activities, or interests related to the content of this manuscript. No grants, contracts, financial support, or other forms of support were received for this study.

## Funding

None.

## Ethical approval

Not required.

## Declaration of competing interest

None declared.

## Appendix Supplementary materials

Supplemental Fig. 1. Normal upper eyelid position (top) versus upper eyelid retraction (bottom), demonstrating increased marginal reflex distance 1 (MRD1) and upper scleral show characteristic of the retracted eyelid.
