# Supplementary material for: Upper eyelid retraction: meta-analysis & systematic review of surgical outcomes
Source: JPRAS Open. 2026 Sep 16;52:475–92. doi: 10.1016/j.jpra.2026.09.018 (PMC13634782; doi:10.1016/j.jpra.2026.09.018)
Supplement: Supplementary file 1 [file mmc1.zip › Supplemental Table 2.docx]

**Supplemental Table 2. Surgical Technique Classification**

| **Macro-Area** | **Included Procedures** |
| --- | --- |
| **Blepharotomy** | Full-thickness anterior blepharotomy (graded, modified, septum-preserving variants) |
| **Combined/Hybrid** | Blepharotomy with Müllerectomy; Levator–Müller recession with partial myotomy/lengthening |
| **Levator/Müller recession** | Combined levator aponeurosis and Müller muscle recession (Henderson procedure, transconjunctival or anterior approaches, septum-opening/preserving variants) |
| **Levator-only** | Levator aponeurosis recession (with/without adjustable sutures, skin or conjunctival approach, Harvey–Mourits technique, pre-Whitnall recession) |
| **Müller-only** | Müllerectomy; Müllerotomy with graded disinsertion |
| **Spacer graft** | Levator lengthening with interposition of autologous (deep temporal fascia, orbital fat/septal flap) or non-autologous (human acellular dermal matrix, bovine acellular dermal matrix, banked sclera) spacer materials |

**Search Strategy**

Search strategy (25/08/2025 11:40)

PUBMED: ( (Eyelid OR eyelid* OR palpebr*) AND ("upper eyelid retraction" OR "upper lid retraction" OR (eyelid* AND retract*)) ) AND ( (Levator OR levator recession OR levator lengthening OR levator myotomy OR "hang-back" OR "hang back" OR levator disinsertion OR levator aponeurosis) OR (Müller muscle OR Mueller OR Muller OR Mullerectomy OR müller muscle recession OR muller muscle excision OR muller disinsertion) OR (blepharotomy OR blepharotom* OR "full-thickness blepharotomy" OR "graded blepharotomy" OR "anterior blepharotomy" OR "partial-thickness blepharotomy") OR ((spacer OR graft* OR interposition) AND ("fascia lata" OR fascia OR sclera* OR "hard palate" OR "acellular dermal matrix" OR ADM OR "dermis fat" OR "dermis-fat" OR mersilene)) OR ("adjustable suture*" OR "anterior approach" OR "posterior approach") )

411 results

Cochrane: ("upper eyelid retraction" OR "upper lid retraction" OR "eyelid retraction" OR (eyelid retract*)) AND ( "levator recession" OR "levator lengthening" OR "levator myotomy" OR "levator aponeurosis" OR "levator disinsertion" OR "hang back" OR "hang-back" OR "muller muscle" OR "müller muscle" OR mueller OR muller OR mullerectomy OR "muller muscle recession" OR "muller muscle excision" OR blepharotomy OR blepharotom* OR "full-thickness blepharotomy" OR "graded blepharotomy" OR "anterior blepharotomy" OR "partial-thickness blepharotomy" OR ((spacer OR graft* OR interposition) OR ("fascia lata" OR fascia OR sclera* OR "hard palate" OR "acellular dermal matrix" OR ADM OR "dermis fat" OR mersilene)) OR "adjustable suture*" OR "anterior approach" OR "posterior approach" )

25 Trials matching


Scopus: ( "upper eyelid retraction" OR "upper lid retraction" OR "eyelid retraction" OR ( eyelid AND retract* ) ) AND ( "levator recession" OR "levator lengthening" OR "levator myotomy" OR "levator aponeurosis" OR "levator disinsertion" OR "hang back" OR "hang-back" OR "muller muscle" OR "müller muscle" OR mueller OR muller OR mullerectomy OR "muller muscle recession" OR "muller muscle excision" OR blepharotomy OR blepharotom* OR "full-thickness blepharotomy" OR "graded blepharotomy" OR "anterior blepharotomy" OR "partial-thickness blepharotomy" OR ( "spacer" OR "graft*" OR "interposition" ) AND ( "fascia lata" OR fascia OR sclera* OR "hard palate" OR "acellular dermal matrix" OR ADM OR "dermis fat" OR mersilene ) OR "adjustable suture*" OR "anterior approach" OR "posterior approach" )

**Standardized conversion rules for margin reflex distance 1 (MRD1):**

Assuming a normal palpebral fissure height (PFH) of approximately 10 mm (MRD1 + MRD2), and a physiological MRD1 of 4.0 mm in Caucasian^1,2^ and 3.5 mm in Asian populations ^2,3^, MRD1 was estimated as MRD1_normal + (PFH − 10)/2, reflecting that approximately half of the change in fissure height occurs at the upper eyelid margin. When scleral show or retraction above the limbus was reported, the measured value was added directly to the normal MRD1.

1.	Rana K, Beecher MB, Caltabiano C, Zhao Y, Verjans J, Selva D. Normal periocular anthropometric measurements in an Australian population. *Int Ophthalmol*. 2023;43(8):2695-2701. doi:10.1007/s10792-023-02669-3

2.	Murchison AP, Sires BA, Jian-Amadi A. Margin Reflex Distance in Different Ethnic Groups. *Arch Facial Plast Surg*. 2009;11(5):303. doi:10.1001/archfacial.2009.9

3.	Suhk JH, Kiranantawat K, Nguyen AH. Physical Evaluation of the Asian Blepharoplasty Patient. *Semin Plast Surg*. 2015;29(3):145-157. doi:10.1055/s-0035-1556851
