# Supplementary material for: Upper eyelid retraction: meta-analysis & systematic review of surgical outcomes
Source: JPRAS Open. 2026 Sep 16;52:475–92. doi: 10.1016/j.jpra.2026.09.018 (PMC13634782; doi:10.1016/j.jpra.2026.09.018)
Supplement: Supplementary file 1 [file mmc1.zip › Supplemental Table 3.docx]

**Supplemental Table 3**

Overcorrection Rates by Technique

| **Technique*** | **k** | **Events/Total** | **Pooled %** | **95% CI** | **I²** | **95% PI** | **p-value** |
| --- | --- | --- | --- | --- | --- | --- | --- |
| Blepharotomy | 7 | 5/335 | 2.68 | 1.91-3.76 | 0% | 0.87-5.39 | 0.99 |
| Levator-only | 11 | 36/420 | 10.8 | 7.63-14.97 | 4.9% | 7.05-14.05 | 0.40 |
| Levator/Müller R-R | 14 | 61/1223 | 5.30 | 2.70-10.17 | 68.7% | 0.53-30.21 | <0.0001 |
| Spacer graft | 11 | 20/483 | 6.31 | 3.20-12.04 | 43.6% | 1.11-21.7 | 0.06 |
| Combined/hybrid | 3 | 6/262 | 3.10 | 1.04-8.85 | 0% | 0.50-13.40 | 0.62 |

*R-R = recession-resection; PI = prediction interval; I² = heterogeneity index*

*Muller-only procedures not evaluated due to insufficient data (k=2, n=36), Unfolding release technique by Zhou not evaluated
