# Supplementary material for: Upper eyelid retraction: meta-analysis & systematic review of surgical outcomes
Source: JPRAS Open. 2026 Sep 16;52:475–92. doi: 10.1016/j.jpra.2026.09.018 (PMC13634782; doi:10.1016/j.jpra.2026.09.018)
Supplement: Supplementary file 1 [file mmc1.zip › Supplemental Table 4.docx]

**Supplemental Table 4**

Undercorrection Rates by Technique

| **Technique** | **k** | **Events/Total** | **Pooled %** | **95% CI** | **I²** | **95% PI** | **p-value** |
| --- | --- | --- | --- | --- | --- | --- | --- |
| Blepharotomy | 7 | 19/335 | 7.8 | 4.8-12.3 | 0% | 4.14-11.89 | 0.50 |
| Levator-only | 11 | 23/420 | 7.1 | 5.3-9.5 | 0% | 3.94-9.51 | 0.88 |
| Levator/Müller R-R | 14 | 110/1223 | 9.8 | 5.7-16.3 | 79.0% | 1.67-37.38 | <0.0001 |
| Spacer graft | 11 | 13/483 | 4.3 | 1.9-9.4 | 44.6% | 0.61-22.07 | 0.054 |
| Combined/hybrid | 3 | 8/262 | 3.8 | 0.1-58.8 | 79.9% | 0.02-88.33 | 0.0068 |

*R-R = recession-resection; PI = prediction interval; I² = heterogeneity index*

*Muller-only procedures not evaluated due to insufficient data (k=2, n=36), Unfolding release technique by Zhou not evaluated
