# Supplementary material for: Upper eyelid retraction: meta-analysis & systematic review of surgical outcomes
Source: JPRAS Open. 2026 Sep 16;52:475–92. doi: 10.1016/j.jpra.2026.09.018 (PMC13634782; doi:10.1016/j.jpra.2026.09.018)
Supplement: Supplementary file 1 [file mmc1.zip › supplemental table 5.docx]

Supplemental Table 5. Risk of Bias

| **Symbol** | **RoB 2 (RCTs)** | **ROBINS-I (NRS)** | **Interpretation** |
| --- | --- | --- | --- |
| **+** | Low risk | Low | Bias unlikely to alter results |
| **?** | Some concerns | Moderate | Bias may alter results |
| **−** | High risk | Serious | Bias likely to alter results |

# Results

## Randomized Controlled Trials (n=2)

**Table S1.** Risk of bias assessment for randomized controlled trials using RoB 2. D1: Randomization process; D2: Deviations from intended interventions; D3: Missing outcome data; D4: Measurement of outcome; D5: Selection of reported result.

| **Study** | **D1** | **D2** | **D3** | **D4** | **D5** | **Overall** |
| --- | --- | --- | --- | --- | --- | --- |
| Gonçalves 2017 | ? | + | + | + | ? | ? |
| Ueland 2024 | ? | + | + | ? | + | ? |

## Non-Randomized Studies (n=37)

**Table S2.** Risk of bias assessment for non-randomized studies using ROBINS-I. D1: Confounding; D2: Selection of participants; D3: Classification of interventions; D4: Deviations from intended interventions; D5: Missing data; D6: Measurement of outcomes; D7: Selection of reported results.

| **Study** | **D1** | **D2** | **D3** | **D4** | **D5** | **D6** | **D7** | **Overall** |
| --- | --- | --- | --- | --- | --- | --- | --- | --- |
| Looi 2006 | ? | ? | + | + | ? | + | + | ? |
| Khatavi 2017 | ? | + | + | + | + | + | ? | ? |
| Mourits 1999 | − | + | + | + | ? | ? | ? | − |
| Zhou 2025 | − | ? | + | + | ? | + | ? | − |
| Ben Simon 2005 (Ophthalmology) | ? | ? | + | + | + | ? | + | ? |
| Ben Simon 2005 (AJO) | ? | + | + | + | + | ? | + | ? |
| Sun 2018 | ? | + | + | + | ? | ? | + | ? |
| Ceisler 1995 | ? | ? | + | + | ? | ? | + | ? |
| Chalfin & Putterman 1979/1981 | − | + | + | + | ? | + | + | − |
| Cho 2012 | − | ? | + | ? | ? | ? | + | − |
| Schwarz & Spinelli 2008 | ? | ? | + | ? | + | ? | + | ? |
| Dixon 1982 | − | ? | ? | − | ? | ? | ? | − |
| Doxanas & Dryden 1981 | ? | ? | + | ? | + | ? | + | ? |
| Park 2025 | ? | ? | + | ? | ? | ? | + | ? |
| Evans 2017 | ? | + | + | + | + | + | + | ? |
| Hintschich & Haritoglou 2005 | ? | ? | + | + | ? | ? | ? | ? |
| Demirci 2007 | ? | ? | + | + | + | ? | + | ? |
| Elner 2003 | ? | ? | + | + | ? | ? | + | ? |
| Guastella 2022 | + | + | + | + | + | + | + | + |
| Hassan 2005 | − | ? | ? | ? | ? | ? | + | − |
| Olver & Fells 1995 | − | ? | + | + | ? | ? | + | − |
| Vu 2022 | ? | + | + | + | + | + | + | ? |
| Lee 2015 (PRS) | − | − | + | ? | ? | + | + | − |
| Lee 2018 (Orbit) | ? | ? | + | + | ? | ? | ? | ? |
| Soares & França 1991 | ? | ? | + | + | ? | ? | ? | ? |
| Ueland 2014 | ? | ? | + | + | ? | ? | ? | ? |
| Mourits & Koornneef 1991 | ? | ? | + | + | ? | ? | ? | ? |
| McNab 2004 | ? | ? | + | + | ? | ? | ? | ? |
| Nimitwongsakul 2013 | ? | ? | + | + | ? | ? | ? | ? |
| Lee 2015 (Ann Plast Surg) | ? | ? | + | + | + | ? | ? | ? |
| Lee 2019 | + | + | + | + | + | + | + | + |
| Pinas 2020 | ? | + | + | + | + | + | + | ? |
| Sales-Sanz 2023 | + | + | + | + | + | + | + | + |
| Shortt 2011 | ? | + | + | + | ? | + | + | ? |
| Small 1995 | ? | + | + | + | ? | + | + | ? |
| Watanabe 2013 | ? | + | + | + | + | + | + | ? |
| Woog 1996 | ? | + | + | + | + | + | + | ? |
